# Multi-omics for studying and understanding polar life

**DOI:** 10.1038/s41467-023-43209-y

**Published:** 2023-11-17

**Authors:** M. S. Clark, J. I. Hoffman, L. S. Peck, L. Bargelloni, D. Gande, C. Havermans, B. Meyer, T. Patarnello, T. Phillips, K. R. Stoof-Leichsenring, D. L. J. Vendrami, A. Beck, G. Collins, M. W. Friedrich, K. M. Halanych, J. F. Masello, R. Nagel, K. Norén, C. Printzen, M. B. Ruiz, S. Wohlrab, B. Becker, K. Dumack, F. Ghaderiardakani, K. Glaser, S. Heesch, C. Held, U. John, U. Karsten, S. Kempf, M. Lucassen, A. Paijmans, K. Schimani, A. Wallberg, L. C. Wunder, T. Mock

**Affiliations:** 1 British Antarctic Survey, UKRI-NERC, High Cross, Madingley Road, Cambridge, CB3 0ET UK; 2 Universität Bielefeld, VHF, Konsequenz 45, 33615 Bielefeld, Germany; 3 Department of Comparative Biomedicine and Food Science, Università degli Studi di Padova, Viale dell’Università 16, I-35020 Legnaro, Italy; 4 Microbial Ecophysiology Group, Faculty of Biology/Chemistry & MARUM, University of Bremen, Leobener Straße 3, 28359 Bremen, Germany; 5 Alfred-Wegener-Institut Helmholtz-Zentrum für Polar- und Meeresforschung, Am Handelshafen 12, 27570 Bremerhaven, Germany; 6 Institute for Chemistry and Biology of the Marine Environment, University of Oldenburg, Oldenburg, Germany; 7 Helmholtz Institute for Functional Marine Biodiversity at the University of Oldenburg (HIFMB), 23129 Oldenburg, Germany; 8 Alfred-Wegener-Institute Helmholtz Centre for Polar and Marine Research, 14473 Potsdam, Germany; 9 Staatliche Naturwissenschaftliche Sammlungen Bayerns, Botanische Staatssammlung München (SNSB-BSM), Menzinger Str. 67, 80638 München, Germany; 10 Senckenberg Biodiversity and Climate Research Centre & Loewe-Centre for Translational Biodiversity Genomics, Senckenberganlage 25, 60325 Frankfurt am Main, Germany; 11 Center for Marine Science, University of North Carolina, 5600 Marvin K. Moss Lane, Wilmington, NC 28409 USA; 12 Justus-Liebig-Universität Gießen, Giessen, Germany; 13 Department of Zoology, Stockholm University, 106 91 Stockholm, Sweden; 14 Natural History Museum Frankfurt, Senckenberganlage 25, 60325 Frankfurt am Main, Germany; 15 Universität Duisburg-Essen, Universitätstrasse 5, 45151 Essen, Germany; 16 Universität zu Köln, Institut für Pflanzenwissenschaften, Zülpicher Str. 47b, 60674 Köln, Germany; 17 Universität zu Köln, Terrestrische Ökologie, Zülpicher Str. 47b, 60674 Köln, Germany; 18 Institute for Inorganic and Analytical Chemistry, Friedrich Schiller University Jena, Lessingstraße 8, 07743 Jena, Germany; 19 Institute of Biological Sciences, Applied Ecology and Phycology, University of Rostock, Albert-Einstein-Straße 3, 18059 Rostock, Germany; 20 Botanischer Garten und Botanisches Museum Berlin, Freie Universität Berlin, Königin-Luise-Straße 6-8, 14195 Berlin, Germany; 21 Department of Medical Biochemistry and Microbiology, Uppsala University, Husargatan 3, 751 23 Uppsala, Sweden; 22 School of Environmental Sciences, University of East Anglia, Norwich Research Park, Norwich, NR4 7TJ UK; 23 Present Address: Manaaki Whenua—Landcare Research, 231 Morrin Road St Johns, Auckland, 1072 New Zealand; 24 Present Address: School of Biology, University of St Andrews, St Andrews, Fife, KY16 9TH UK

**Keywords:** Conservation genomics, Molecular ecology

## Abstract

Polar ecosystems are experiencing amongst the most rapid rates of regional warming on Earth. Here, we discuss ‘omics’ approaches to investigate polar biodiversity, including the current state of the art, future perspectives and recommendations. We propose a community road map to generate and more fully exploit multi-omics data from polar organisms. These data are needed for the comprehensive evaluation of polar biodiversity and to reveal how life evolved and adapted to permanently cold environments with extreme seasonality. We argue that concerted action is required to mitigate the impact of warming on polar ecosystems via conservation efforts, to sustainably manage these unique habitats and their ecosystem services, and for the sustainable bioprospecting of novel genes and compounds for societal gain.

## Abstract (web summary)

Endangered polar ecosystems play critical roles in the Earth’s climate system and comprise many different habitats with unique organisms. Here, the authors propose a community road map to use multi-omics data from polar organisms for conservation, ecosystem services and societal gain.

## Introduction

**Fig. 1:**
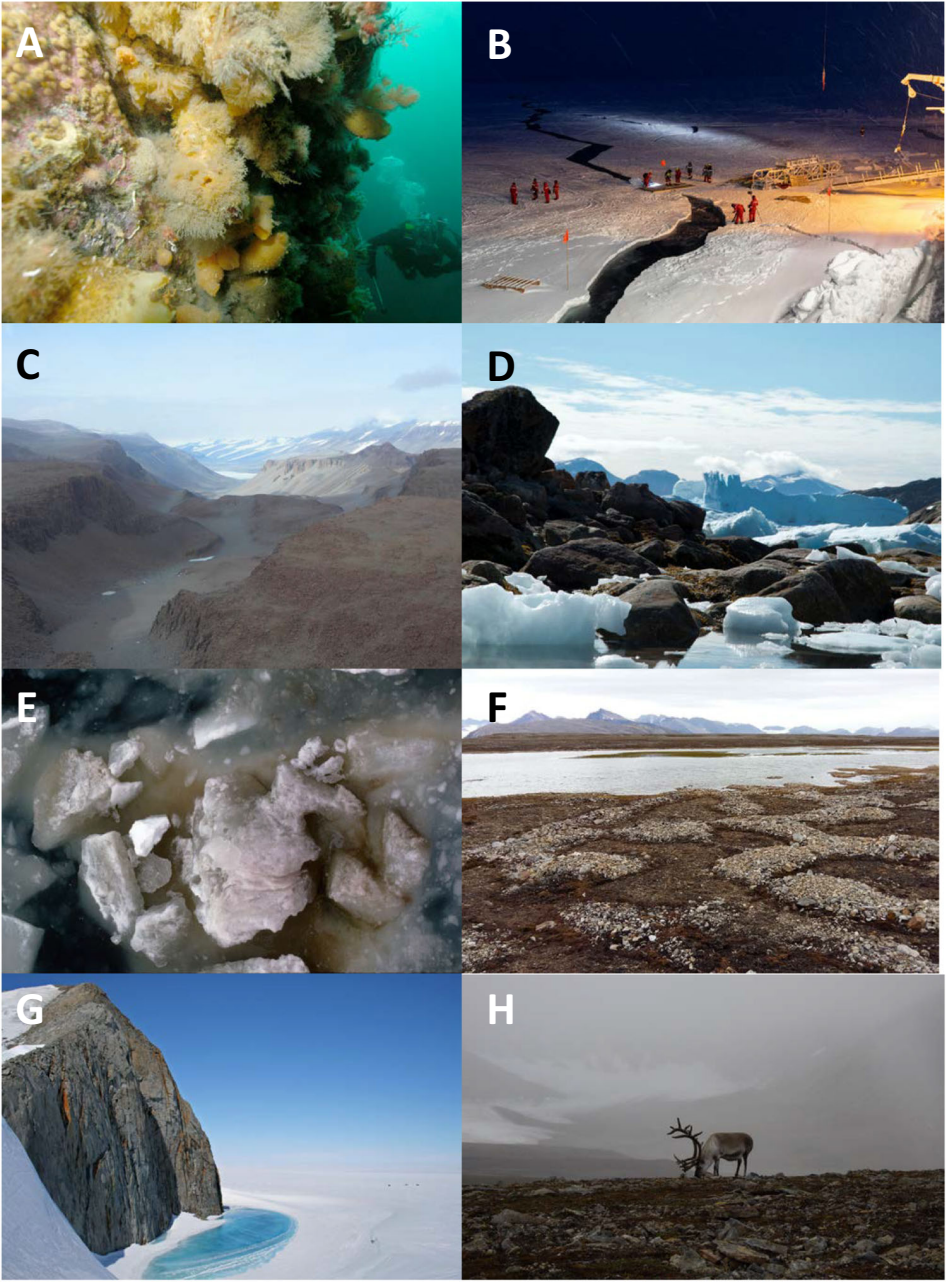
Different polar environments. **A** Scientific divers inspect benthic communities on a submerged wall on Anchorage Island, near Rothera Research Station on the Antarctic Peninsula. Photo from BAS photo library. Photographer John Withers. **B** The Arctic winter on the MOSAiC expedition, during which the German research icebreaker Polarstern spent a year drifting through the Arctic Ocean trapped in the ice (https://mosaic-expedition.org/). Photographer Marcel Nicolaus, Alfred Wegener Institute for Polar and Marine Research, Bremerhaven, Germany. **C** McMurdo Dry Valleys in Victoria Land, East Antarctica. These valleys are an unusual region of extremely low humidity without snow or ice cover, which have not seen precipitation for ~2 million years. Photo from BAS photo library. Photographer John Shears. **D** Arctic intertidal region near Upernavik, Greenland. Intertidal species include blue mussels (*Mytilus edulis*) and macroalgae. Photographer Jakob Thyrring. **E** Sea ice upturned by the ice strengthened research vessel RRS Bransfield in the Weddell Sea, Antarctica exposing ice algae growing on and within the underside of the sea ice. Ice algae are an abundant source of food for overwintering krill. Photo from BAS photo library. Photographer Chris Gilbert. **F** Arctic permafrost in Svalbard dominated by biological soil crusts with characteristic geomorphic feature of tundra polygons. Photographer Svenja Heesch, University of Rostock. **G** Nunatak on the northern Churchill Peninsula (Oscar II Coast, Graham Land) and frozen freshwater melt pool. Photo from BAS photo library. Photographer Teal Riley. **H** Arctic tundra on Svalbard with endemic reindeer. Photographer Melody Clark (BAS). All photographs published with permission and all supporting imagery from the BAS Image Collection is published according to the image rights agreement between each photographer and the British Antarctic Survey.

**Fig. 2:**
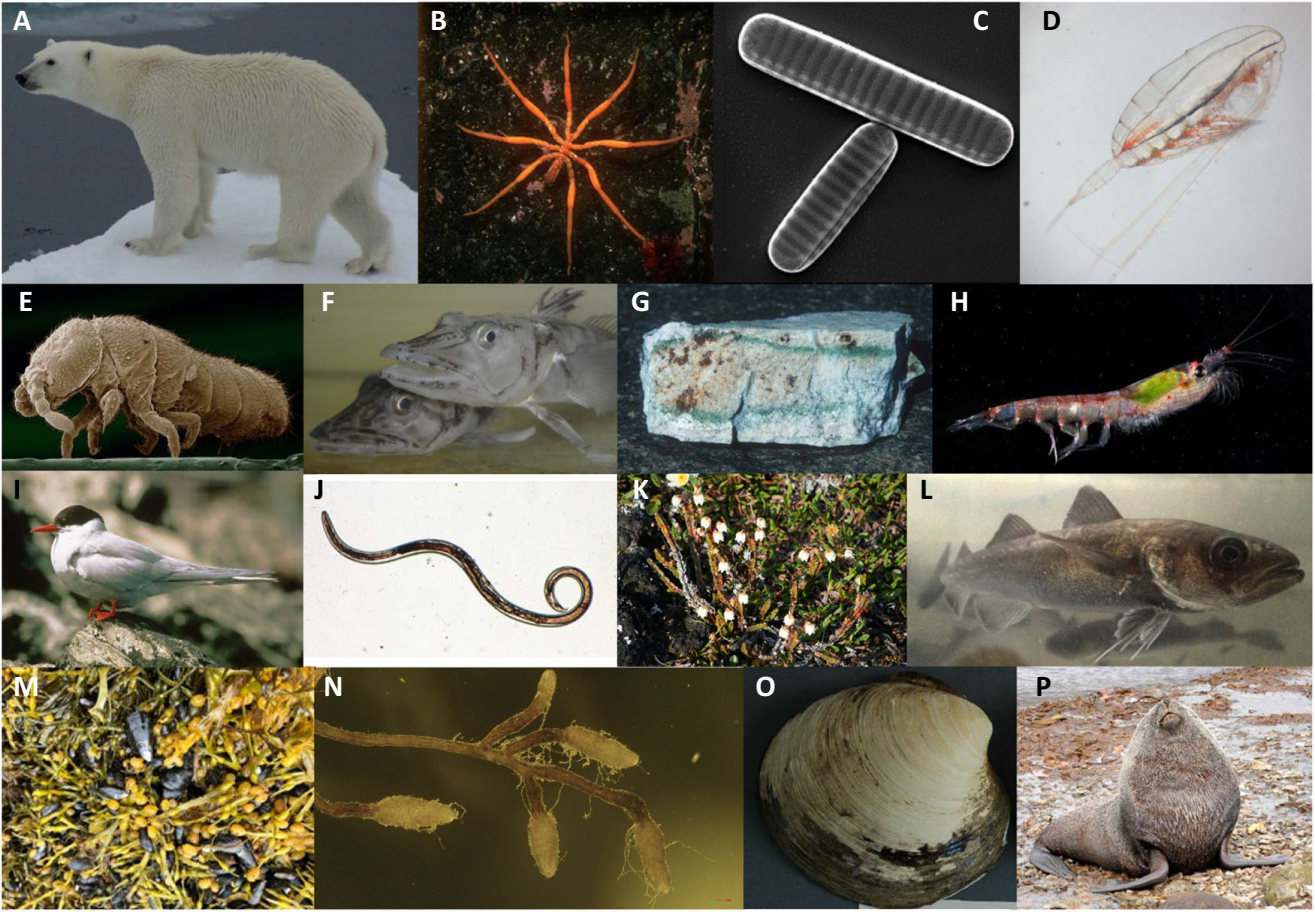
Examples of polar species with unique adaptations. More extensive details of adaptations with associated references in Supplementary Note 2. **A** Polar bears have a modified cardiovascular system allowing them to tolerate chronically elevated levels of serum cholesterol in their diet. Photo from BAS photo library. Photographer Angelika Renner. **B** Antarctic sea spiders are examples of polar gigantism. Photo from BAS photo library. Photographer Dave Bowden. **C** Antarctic diatoms produce ice antifreeze proteins to survive in sea ice. Photograph from Thomas Mock, University of East Anglia. **D** Copepods accumulate lipids (up to 70% of individual dry weight) to survive Arctic winters. Photograph from Kim Last, Scottish Association for Marine Sciences. **E** Antarctic springtails survive down to −30 °C via rapid cold hardening. Photo from BAS photo library. Photographer Pete Bucktrout. **F** Icefish are the only vertebrates that lack haemoglobin. Photograph from Gianfranco Santovito, University of Padua. **G** Antarctic endolithic communities in rock survive the most extreme conditions. Photo from BAS photo library. Photographer David Wynn-Williams. **H** Krill has the largest biomass of any wild animal on the planet. Photo from BAS photo library. Photographer Pete Bucktrout. **I** Arctic tern undertakes the longest migration on Earth. Photo from BAS photo library. Photographer Callan Duck. **J** Antarctic nematodes normally live in temperatures of down to −7 °C, but some can survive at −80 °C. Photograph from Kevin Newsham, British Antarctic Survey. **K** Arctic Bell-heather thrives in deep snow over winter. Photographer from Melody Clark, British Antarctic Survey. **L** Polar cod shows convergent evolution of antifreeze glycoprotein to survive the cold. Photograph from Till Luckenbach, Helmholtz Centre for Environmental Research—UFZ. **M** Blue mussels and macroalgae can survive to 36 °C in the Greenland intertidal. Photographer Jakob Thyrring, Aarhus University. **N** Ectomycorrhiza colonise plant roots and play vital roles in protecting the plant from extreme conditions. Photographer Kevin Newsham, British Antarctic Survey. **O** An Ocean quahog holds the record of the longest-lived animal on Earth. Photographer Al Wanamaker, Iowa State University. **P** Antarctic fur seal genomics is revealing signals of past hunting pressures. Photographer Joseph Hoffman, University of Bielefeld. All photographs published with permission and all supporting imagery from the BAS Image Collection is published according to the image rights agreement between each photographer and the British Antarctic Survey.

Polar regions comprise most of the world’s cryosphere. They play critical roles in the Earth’s climate system and global nutrient circulation and comprise many different habitats with unique organisms (Figs. 1 and 2). The Southern Ocean accounts for ~40% of the global oceanic uptake of anthropogenic CO_2_ and ~50% of the total atmospheric uptake^1^ and is essential for the provision of nutrients that sustain oceanic productivity globally^2^. Critical to polar ecosystem processes are the endemic biota, from viruses to megafauna. Polar biodiversity and ecosystem functioning are under threat due to anthropogenic climate change. In the Arctic, temperatures are rising rapidly^3^, destabilising the Arctic jet stream and increasing the likelihood of extreme weather events in temperate regions^4^. Warming-induced retreat and thinning of the pan-Arctic sea-ice is increasing the influx of waters from surrounding seas into the Arctic Ocean (a process often termed “Atlantification”)^5^. On land, permafrost melting and collapsing Arctic coastlines are altering ecological interactions and biogeochemistry^6,7^. The Antarctic Peninsula has already experienced substantial levels of warming^8^. Overall, areas with an annual mean near-surface air temperature (at 1.5–2 m above the surface) below −20 °C are projected to shrink by ~7% by 2099 in CMIP6 climate models forced by the SSP2-4.5 (mid-range) scenario and by ~14% in CMIP6 models forced by the SSP3-7.0 (high emissions) scenario (Supplementary Note 1).

Climate-induced changes in the polar regions are already altering species distributions on land and in the sea, with major impacts on ecosystem function^9^. Some species have shifted polewards, for example the *Calanus* (marine copepod) complex and their predators including cod and herring, with important economic consequences^10^. Similarly, the southward shift of Antarctic krill has driven widespread changes from a krill-based to a salp-based gelatinous ecosystem^11^, affecting commercial krill fisheries and predators including penguins, seals, and whales^12^. In parallel, polar terrestrial species are affected by multiple, interacting factors (including temperature, water availability, wind patterns, snow and ice cover), confounding our ability to predict large-scale community changes and their consequences^13,14^. Although both polar regions are characterised by extremely low temperatures, the effects of climate change on their ecosystems will differ substantially due to their contrasting geographies and evolutionary histories. Antarctica emerged from the breakup of Gondwana in the Cretaceous period (120 Ma BP), separating from South America ~31 Ma BP. This marked the first onset of major cooling and appearance of sea ice^15^ and initiated the formation of the Antarctic Circumpolar Current and the Antarctic Polar Front, which act as significant barriers to colonisation from lower latitudes. The Arctic has a relatively young biota (post Pleistocene, ~2.58 Ma BP with perennial sea ice occurring ~0.7–2 Ma BP), with few described endemics^16^ (e.g., the polar bear, narwhal, lemming, and plants such as *Saxifraga svalbardensis* and *Draba nivalis*). Furthermore, Arctic ecosystems are contiguous with land masses and continental margins stretching to the tropics, whereas Antarctica has no land or continental shelf links to lower latitudes^17^. Although some taxa have colonised Antarctica after its separation from South America^18^, most of its terrestrial species are Gondwana relicts that found refuge in areas that remained ice-free e.g., geothermal areas or isolated mountain peaks^19^. Hence, the Antarctic largely contains a geographically isolated biota that has evolved in the cold since ~10 Ma BP^20^, whilst the Arctic biota has only experienced extreme cold for ~3 Myr and remains more geographically connected to the rest of the planet.

The most recent IPCC reports (https://www.ipcc.ch/) recognised a key knowledge gap in polar ecosystems. Reliable biodiversity projections for the polar regions can only be achieved with a sufficiently profound understanding of the diversity, ecological functions, and interrelations of polar organisms as well as their resilience to climate change. However, long-term (decadal plus) studies correlating species distributions and abundances to environmental data are scarce in the polar regions. These studies also tend to focus on single species abundances and distributions, such as krill, salps and fur seals in the Southern Ocean^11,12^ and Arctic foxes, voles and lemmings in the Arctic^21,22^. These studies alone cannot produce general insights given the heterogeneous nature of different polar habitats (e.g., sea ice, permafrost, melt ponds, cryoconite holes, ice-free ocean, snow, glaciers and terrestrial habitats (e.g. see Fig. 1), which likely drives local adaptation and ultimately speciation. Furthermore, due to the difficulties in accessing and carrying out research in such extreme environments, particularly in winter, the availability of monitoring programmes to assess changes in distributions of species and their populations, including alterations of gene flow and climate-driven range shifts, is very limited and is subject to sampling bias towards the most easily accessed sites^23^. Therefore, complementary approaches are required not only to generate predictive future ecosystem biodiversity scenarios, but also to characterise polar biodiversity from a functional perspective and provide insight into evolution and adaptation to the cold. In this Perspective, we champion the use of multi-omics approaches to understand polar ecosystems and adaptation to life in the cold. In this context, we use multi-omics in terms of leveraging data from different omics techniques including genomics (the study of DNA), epigenomics (temporary modifications of DNA), transcriptomics (or gene expression analyses using RNA or cDNA), proteomics (analyses of protein sequence and structure) and metabolomics (biochemical analyses of small molecules such as carbohydrates and amino acids). We propose that multi-omics can contribute to the aforementioned objectives and discuss the main opportunities and challenges.

### The polar omics challenge

The application of omics approaches to polar biodiversity has been the subject of several international publications, including two US NAS/NRC study reports and a multinational Antarctic Horizon Scan study^24–26^. However, the promised omics revolution in polar science has largely failed to materialise. We argue that one reason is rooted in the over-arching governance structures of the polar regions and the advisory role polar science plays in environmental regulation in these regions, which has effectively led to an inward, rather than outward looking science community (Box 1: Polar “silos”). Furthermore, bilateral or multilateral agreements at a political level are required to promote international scientific cooperation for sharing costs and research infrastructure. Polar science programmes are expensive to operate and maintain, with the majority of recent funding targeted at the polar regions going towards logistics and infrastructure, such as new ships and research stations. In addition, most current funding mechanisms for conducting polar research do not operate across national boundaries. Thus, we need “bridge building” based on international agreements including joint funding for research and not only to support infrastructure. Although the actual number of scientists working in the polar regions is not insubstantial, especially when including logistics support staff, this community is fragmented, spread across many different countries, and lacks critical regional mass. The short-term and uncertain nature of national polar biology science funding, accompanied by an insufficient level of international funding agreements to generate science in polar regions and the “silo mentality” of the polar community, has likely slowed the advancement of polar biology, especially in the adoption of new techniques such as genomics and multi-omics. We argue that there is also a bias towards funding physical polar research. Although this research contributes significantly to understanding the societal impacts and economic consequences of climate change (sea level rise, climate models etc.), the same argument applies to polar biota, as they underpin ecosystem services such as fisheries and bioprospecting (Box 2: “The polar regions: Large scale projects for business and policy”).

**Fig. 3:**
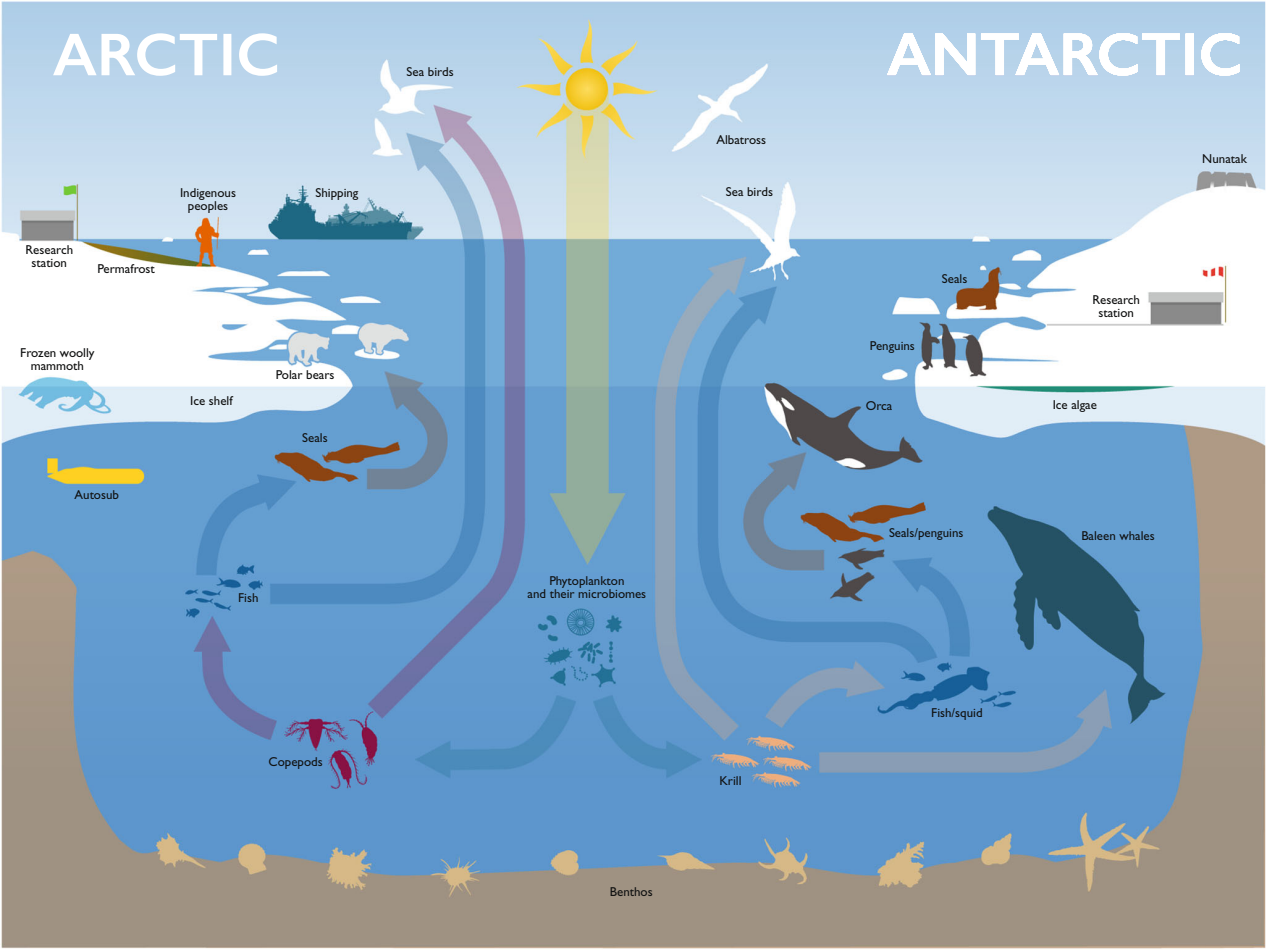
Schematic showing simplified polar ecosystems. Omics can be used to evaluate biodiversity across the whole of the polar Tree of Life from microbes in the ocean, land, ice and permafrost through to the large charismatic mega fauna, such as polar bears, whales, seals and sea birds, as discussed in the main text. Such analyses will reveal adaptations to life in the cold from the single gene to whole animal levels. Furthermore, sequencing multiple individuals in different populations, as represented by the groups of seals, penguins and polar bears will reveal evolutionary histories and population variability, which may provide indications of future resilience. Sequencing of gut contents from any of the species depicted can provide insight into current and changing food webs on land and in the sea. Using the past to predict the future is represented by the frozen woolly mammoth and permafrost. Long-term monitoring for surveillance is represented at both poles by the two research stations, with automatic sampling depicted by the unmanned vehicle autosub. Related to surveillance is the pictogram of the ship, representing the potential introduction of alien invasives, as can be monitored using eDNA.

We argue that polar biological research requires a step change on a collaborative, integrative and international level led by genomics (and multi-omics) approaches, which will produce the data required to address the grand challenges in polar science. Hence, in this Perspective we review areas of polar science where genomics can make a real impact (Fig. 3). Along with examples of the state of the art in each area, we present future perspectives and recommendations for each area, which provide more detail in support of an all-encompassing road map for the next 5–10 years (see “Polar Genomics: a road map”).

Box 1 Polar *“silos”* In 1959, the Antarctic Treaty was signed, designating Antarctica and the Southern Ocean south of 60° as a land for peace and science. Article II of the Treaty promotes freedom of scientific investment and Article III promotes international co-operation in scientific investigation. These founding principles were underpinned by the formation of SCAR (The Scientific Committee on Antarctic Research) to facilitate international collaboration in Antarctic research. In 1996 the Arctic Council, an intergovernmental forum promoting co-operation in the Arctic, was formed. These political and administrative bodies have been very successful in developing and co-ordinating polar science communities. There are regular polar-focussed conferences (e.g. the international SCAR Open Science Conference every 2 years); polar peer-reviewed journals, such as Polar Research, Antarctic Science, Polar Biology and Arctic, Antarctic and Alpine Research; and polar-specific databases (e.g. SCAR-MarBIN (SCAR Marine Biodiversity Information Network) (https://www.scarmarbin.be), Southern Ocean mollusc database (SOMBASE) (www.antarctica.ac.uk/SOMBASE), Arctic Ocean Diversity (http://www.arcodiv.org/)). Whilst such co-ordination of data and research is useful, we argue that it has also led to an inward-looking community, with polar research viewed as “separate” and “special” by both those involved and the wider scientific community. This is reflected in the slow and disparate adoption of molecular techniques and the rather low impact factors of polar journals (~1.8–2.4), which indicate relatively low citation rates and a lack of wider interest beyond the polar community. Engagement with the wider community is essential. To increase visibility and interactions with other scientific fields, we need to identify globally important questions in ecology and evolution that can be addressed using polar species as exemplars. With wider engagement, it is also easier to keep up to date with the latest cutting-edge methodologies and form cross-disciplinary collaborations. Without wider engagement, the biological polar science community will not attain sufficient critical mass for large-scale funding initiatives.

Box 2 The polar regions: large-scale projects for business and policy In the current climate crisis, a key driver for science is economic impact. In this respect, physics-based projects have a distinct advantage as evidenced by recent funding allocations to, for example, drilling for the oldest ice core (https://www.beyondepica.eu/en/) and the Greenland Ice Core Projects (https://eastgrip.nbi.ku.dk/). These projects analyse climate variability over multiple glacial cycles to investigate climate forcing for input into the latest climate models. SOCCUM (Southern Ocean Carbon and Climate Observations and Modelling project) (https://soccom.princeton.edu/) is an oceanographic consortium studying the influence of the Southern Ocean on the global climate. Projects such as the Greenland Ice Sheet Project (https://climatechange.umaine.edu/gisp2/) and the International Thwaites Glacier Collaboration (https://thwaitesglacier.org/) study ice sheet/shelf instability and their contribution to sea level rise, data which feed directly into flood mitigation strategies and insurance company calculations of future risks. At first glance, the outputs of polar biology have less tangible associated economics, despite expected future losses of polar biodiversity and regime shifts, which will lead to global food security problems. In the Arctic, loss of biodiversity will deleteriously impact Indigenous people and their traditional livelihoods. Shifting fish and krill distributions threaten the viability of international commercial fisheries at a cost of hundreds of millions of dollars every year. Polar species are also reservoirs of novel proteins, as well as known proteins, that work more effectively at lower temperatures. Identification of polar novel variants of industrial enzymes that work at lower temperatures to those currently in use could massively reduce energy budgets worldwide to meet Net Zero. We need to emphasise much more strongly that diminishing biodiversity carries massive costs to human societies with figures associated to these costs. We also need to recognise the value of polar natural resources to provide future solutions to global problems to incentivise the funding of polar biology.

### Genome evolution in response to life in the cold

Polar ecosystems are characterised by extreme environmental conditions and intense seasonality. Polar seas rarely reach temperatures above 5 °C, whilst terrestrial and some highly specialised habitats, such as cryoconite holes and endoliths, are strongly impacted by seasonal freeze-thaw processes, requiring the biota to possess highly specialised physiological abilities to persist and thrive (for some examples, see Fig. 2 and Supplementary Note 2). The difference between the extreme isolation in Antarctica and the strong connectedness to tropical latitudes in the Arctic has produced very different levels of endemicity including the evolution of new species that cannot persist outside polar ecosystems (e.g., psychrophiles (for some examples, see Fig. 2)). The most comprehensive way to characterise the vast diversity of polar organisms and to understand their biological differences is via comparative evolutionary genome and metagenome analyses. An important step towards this goal is to assemble collections of representative reference genomes including metagenome assembled genomes, which provide far more fine detail on genetic adaptation and evolution than analysis of, for example, single barcodes or candidate gene approaches.

#### State of the art

There are more than 130 genomes available from psychrophilic bacteria and archaea and ~800 metagenome-assembled genomes have recently been added from the Arctic Ocean^27,28^. In contrast to microbial genomics, the sequencing of genomes from multicellular polar species has lagged behind, although significant numbers of transcriptomes are already available. To date, reference genomes are only available for a handful of species, including charismatic megafauna such as polar bears, penguins, Antarctic fur seals, Arctic foxes and narwhals^29–33^, with additional genomes having been sequenced from a number of polar fishes^34,35^ and the midges *Belgica antarctica* and *Parachlus steinenii*^36,37^. Although crustaceans and other invertebrates play important roles by linking primary producers to tertiary consumers such as birds, seals and whales, to date only mitochondrial genomes are available for a handful of these key species, the exception being the recent publication of the Antarctic krill genome (*Euphasia superba*)^38^.

The first genomes and metagenomes from polar microbial communities have not only provided insights into their diversity, but also into how they might respond to environmental changes such as warming. Furthermore, the power of whole genome sequencing can be amplified if efforts are concentrated on a range of related species or multiple members of the same species. For example, this approach has uncovered signals of positive selection in genes implicated in cold adaptation in penguins and the ice fish *Chionodraco hamatus*^31,39^. Transposon expansion has also been shown to have likely played a role in the evolution of genomic regions incorporating Antarctic-specific traits and derived characteristics, such as antifreeze glycoproteins and haemoglobin loss in Notothenioid fish^35^ and that zinc requirements have driven the evolution of polar phytoplankton^40^. As another example, genome sequencing has uncovered a reorganisation of the cardiovascular system of polar bears that allows them to tolerate chronically elevated levels of serum cholesterol in their diet^41^.

#### Future perspectives and recommendations

As a much more comprehensive set of genomes than currently available is required to understand how polar species have adapted to life in the cold, this should become a funding priority. A particularly powerful approach is to exploit comparative genomics approaches to analyse taxa that occur at both poles, such as krill, the pteropod *Clione*, the anemone *Dactylanthus* or diatoms, to identify evolutionary adaptations to the cold while effectively controlling for biogeographic history and genetic isolation. In addition, RNA-seq, ChIP-seq, ATAC-seq and DAP-seq methods should be fully integrated into genome sequencing projects to help identify regulatory variants and regulatory elements. To date, the nature and role of regulatory elements in polar organisms is poorly understood, yet such elements play significant roles in the evolution and plasticity of important ecological traits in non-polar species^42,43^. Hence, polar genomes likely contain regulatory mechanisms to enable organisms to cope with the extremely variable seasonal conditions in polar habitats, requiring regulatory plasticity beyond that necessary to thrive outside polar ecosystems.

Given the current dramatic rates of regional warming at the poles and therefore a significant likelihood of biodiversity loss, we need to also identify those species, and their associated physiologies, that are most at risk and implement gene banking and ex situ conservation strategies as a matter of urgency^44^. This strategy is needed across a range of organisms from microbes to charismatic polar megafauna because of rapidly disappearing sea ice, permafrost and tundra habitats^44^. Failure to act now will result in a substantial loss of knowledge regarding evolutionary adaptation to the cold and will also reduce the potential for the sustainable exploitation (e.g., bioprospecting) of novel genetic variants that are likely absent from non-polar species. Gene banking will require not only a single individual of a given species, but enough coverage within a species to allow representative intraspecific genetic variation to be characterised and conserved.

### Resilience to change: using genomics to understand population dynamics

Multiple aspects of genetic variation are relevant to species persistence over both the short and long term, including genetic diversity, mutation loads, genetic variance for phenotypic traits, population genetic structure, gene flow and local adaptation. Genetic diversity has been described as “the most fundamental dimension of biodiversity” because it impacts individual fitness, population persistence and evolutionary potential, thereby feeding into higher levels of organisation^45^. Closely related to genetic diversity is the concept of the effective population size (Ne). This key parameter in population genetics can be thought of as reflecting the number of reproducing individuals in an idealised population. Ne is important because it affects the rate at which novel genetic variants arise in a population through mutation, as well as rates of allele frequency change and the loss of alleles through genetic drift, and the efficacy of natural selection. Selection is also important and is most efficient in large populations, where genetic drift is relatively weak, meaning that the most numerous species theoretically have the greatest potential to mount adaptive evolutionary responses to changing selection pressures. However, the evolutionary potential of many long-lived polar organisms remains open to question, even if Ne may be large, due to their very long generation times^44^.

#### State of the art

Metagenomic sequencing of epipelagic plankton from the Tara Oceans Campaign (https://fondationtaraocean.org/en/expedition/tara-oceans/) has demonstrated that polar marine ecosystems are the home of some of the World’s most connected species assemblages^46^. By contrast, a review of genetic studies of Southern Ocean seabirds found that their populations are more structured than was previously assumed based on their high dispersal potential^47^. Furthermore, in the marine realm, eukaryotic population structure is commonplace and appears to be shaped by complex interactions between oceanographic features and life-history variation, with direct developers being more genetically structured than indirect developers, even when screened at a relatively coarse level using Amplified Fragment Length Polymorphisms^48^. Using the far more powerful approach of Restriction site-associated DNA sequencing (RADSeq), “chaotic genetic structure” has been demonstrated in a widespread and highly abundant Antarctic marine invertebrate, (the limpet *Nacella concinna*) suggesting that strong genetic drift during larval stages can markedly decrease genetic diversity^49^. These findings emphasise the importance of obtaining a mechanistic understanding of polar patterns and processes, which can only be thoroughly understood with population-level, genome-wide data for many species.

In addition to genetic diversity and population structure, the effective population size also plays an important role. For example, many polar marine mammals carry genetic signatures of historical population expansions that coincide with the increasing availability of ice-free habitats after the last glacial maximum, as revealed by mitogenome analysis in narwhals^50^. Hence, understanding past demographic changes can help in predicting future trajectories of polar organisms under climate change. A new suite of approaches based on the analysis of the site frequency spectrum^51^ and their application to reduced representation sequencing approaches, such as RADSeq data has particular promise for inferring such changes, as illustrated by a recent study of Antarctic fur seals^52^. In this species, Ne was substantially reduced by commercial harvesting during the eighteenth and nineteenth centuries, but subsequently recovered to around twice that of the pre-sealing population. This supports the “krill surplus hypothesis”, which argues that the removal of the great whales produced a surplus of Antarctic krill that likely allowed populations of other predators such as seals and penguins to increase^53^.

#### Future perspectives and recommendations

We need to improve our understanding of both local adaptation and gene flow in order to predict the resilience of polar ecosystems under future climates. To enable this, comprehensive genome coverage from numerous individuals per species is required, using either reduced representation sequencing approaches or whole genomes alongside cutting-edge bioinformatics approaches. Where available, these data can also be used to investigate genotype-climate associations using approaches including generalised dissimilarity models^54^ and gradient forests^55^. From there, the “genomic offset” can be calculated, a measure of the distance between current variation at putatively adaptive loci and the variation required to maintain the same fitness level under future potential environmental conditions^55^. Another family of approaches includes pedigree-free quantitative genetics^56^ and genomic prediction^57^. The former seeks to estimate trait heritabilities (alongside genetic correlations) by quantifying the strength of association between matrices of genomic relatedness and phenotypic similarity. The latter approach goes a step further by predicting individual breeding values for a given phenotypic trait from genomic data, which then allows the investigation of (potentially cryptic) microevolutionary dynamics^58^.

### Functions and adaptations revealed through omics

Although whole genome studies are extremely insightful in revealing genomic signatures of evolution to life in the cold and their potential to respond to environmental change, ultimately, they are in silico studies, which are descriptive and correlative in nature. Unfortunately, many of the computationally predicted genes in non-model species are annotated as having no associated functional data. Hence, our current understanding of responses to change is perforce constrained to a subset of the most highly conserved genes and the molecular toolbox for functional studies of polar organisms is depauperate.

#### State of the art

Fundamental requirements for functional genomics include not only sequence data, but also the ability to culture organisms, tissues and cells (including the production of immortalised cell lines) under laboratory conditions. These methods should ideally include the development of reverse genetics tools such as genome editing (e.g., CRISPR/Cas9 system), which have the potential to unequivocally determine how genotypes underpin phenotypes exposed to selective forces. However, such culture approaches for tissues and cells are rare for polar species. Currently, microbes such as the psychrophilic bacteria *Colwellia psychrerythraea* 34H and the *Glaciecola psychrophila* strain 170, together with the psychrophilic diatom *Fragilariopsis cylindrus* CCMP1102, might be some of the most well characterised polar organisms for which genomes, transcriptomes, proteomes and physiological data are available to understand how life in the cold evolved. Psychrophilic bacterial genes have also been successfully expressed and characterised in heterologous expression systems such as *Escherichia coli*^59^. *F. cylindrus* has recently become genetically tractable for in vivo studies of gene functions^60^, and together with the Antarctic yeast *Pseudozyma antarctica*, for which CRISPR/Cas9 has recently been employed^61^, these species are the first polar eukaryotes with a molecular toolbox. No such molecular tools are yet available for multicellular polar organisms or their cell lines, although these are essential for the next phase of polar biodiversity research.

Allied to the functional studies detailed above is the biophysical investigation of individual “cold-adapted” proteins. This work is far more advanced in bacteria than any eukaryotic species. For example, specific amino acid substitutions have been identified in microbial genes that increase molecular flexibility and enzyme efficiency in the cold^62^. However, only a handful of biochemical/biophysical studies of cold-adapted Antarctic fish proteins have been described^63^. Even today, it is still virtually impossible to predict how specific amino acid substitutions affect protein function at low temperature^63^ and there is a lack of investigations studying these characteristics at environmental temperatures. The exciting development of sophisticated 3D protein folding prediction algorithms such as Alphafold (https://alphafold.ebi.ac.uk/) shows great promise, although these algorithms are trained on temperate proteins and ultimately produce in silico predicted 3D structures that likely differ from protein conformations at near 0 °C temperatures. In silico approaches can also play a role in deciphering multi-omic functions, for example gene network analyses which incorporate both annotated and non-annotated sequences in the resultant networks^64^.

#### Perspectives and recommendations

To investigate the biophysics of protein functioning in the cold and to characterise unknown gene and protein functions, significant financial and scientific investment is needed to develop tractable polar model systems, particularly for multicellular organisms. This will require technology transfer of cellular functional genomics (including reverse genetics) techniques developed in non-polar model species. In addition, greater use of machine-learning techniques is needed to extract in silico folding data and contextualise cold-adapted amino acid substitutions by mapping amino acid substitutions to 3D structures. Although these approaches may provide clues to protein function, biophysical methods will still be required to robustly validate cold adaptations.

Major gaps still exist in our understanding of genome evolution and how this is linked with adaptations of species in the cold and how this may be influenced by external factors. These knowledge gaps need to be rectified. For example, there are currently virtually no available data for polar organisms on the role of epigenetics, the nature and biological significance of species interactions (including host-microbiome interactions and their contribution to organism health), phenotypic plasticity and how phenotypes are modified across life histories and in response to different environmental conditions. In addition, in the marine realm, many polar species are very long-lived^17^ and it is the older animals that produce the most offspring, yet these older individuals are often the most vulnerable to environmental stressors^65^. Phenotypic plasticity may well act in tandem with reversible epigenetic mechanisms contributing to pliable responses across different environments and changing conditions^66^. However, it is also possible that evolution under stable long-term cold conditions has favoured genetic assimilation, i.e., a reduction of phenotypic plasticity. Polar organisms are therefore highly relevant to the ongoing debate about mutation-led versus plasticity-led evolution. Understanding the nature of genome-level cold adaptation is further complicated when considering polar intertidal and terrestrial species, which are typically exposed to rapid and wide-ranging temperature variations, including extreme cold and the intense seasonality of polar environments^67^. Reproductive cycles, both on land and in the sea, are finely tuned to food and liquid water availability, as well as other environmental conditions such as ice and solar irradiance^67^. In addition, changes in the composition and timing of marine primary producers significantly impact food availability and the survival of marine larvae^17^. How the genome controls responses to intense seasonality is unknown, but such knowledge is integral to understanding how polar species survive in such extreme conditions.

### Predicting the future from the past using omics

Cold conditions in the polar regions facilitate the preservation of DNA, with the oldest records dating back two million years in the Greenland permafrost^68^ and one million years in the Southern Ocean^69^. Fresh water and marine  sediments, permafrosts, soils and other deposits thus act as unique archives of past climate and its effects on ecosystem structure and function. DNA analyses of such samples provide increased taxonomic resolution compared with the challenging taxonomic identification of preserved morphological structures and thus enable more accurate reconstructions of past ecosystems. Whilst these historical samples provide palaeo proxies to reconstruct baselines prior to anthropogenic alterations, they can also be used to predict ecological shifts under current warming^68,70^.

#### State of the art

Ancient DNA (aDNA) approaches include the use of metagenomics for reconstructing sediment communities, mitogenomes and whole genomes. Sedimentary ancient DNA (sedaDNA) studies have already demonstrated shifts in diatom assemblages with past environmental changes, knowledge that will help to improve future ocean and cryosphere risk assessments^70^. Furthermore, a recent metagenomic study of a sediment core in the Bering Sea uncovered a temporal shift from a sea-ice adapted late-glacial ecosystem characterised by diatoms, copepods and codfish, to a warmer, ice-free Holocene ecosystem characterised by cyanobacteria, salmon and herring. This long-term ecosystem shift implies changes in carbon export and benthic food supply in a future scenario in polar seas under enhanced global warming^71^. Moreover, genomic techniques have also successfully reconstructed ancient mitogenomes and whole ancient genomes by using sequence data from the closest extant relatives as scaffolds^72,73^. These approaches have provided improved understanding of the evolution and population history of the extinct megafauna, including past responses to climate change and extinction dynamics in Siberian woolly mammoths and rhinoceroses and Eurasian lemmings^72–74^.

#### Perspectives and recommendations

To understand polar species’ evolutionary potential, we need to reconstruct their histories, ideally by analysing both contemporary and historical samples such as museum collections and ancient DNA in tundra, sediments and ice cores. Palaeogenomics is an expanding field that has made significant methodological advances, including the addition of DNA repair techniques to improve sequencing depth, producing more accurate genome assemblies^75^. Recently, the recovery of ancient metagenome assembled genomes using de novo assembly strategies has expanded the use of aDNA towards the functional profiling of microorganisms from ancient human gut DNA^76^. This offers great promise for extending these approaches to the holobionts of large polar animals encapsulated in permafrost, providing clues to ancient gut diversity, health, diet and past food webs.

### Genomics for surveillance and safeguarding future biodiversity

The current rates of warming in the polar regions are unprecedented. We are seeing responses to climate change in “real-time”. Long-term monitoring programmes that produce multi-decadal datasets provide valuable input to climate models and IPCC reports (https://www.ipcc.ch), which help to inform future policy initiatives. Long-term monitoring sites with their historic datasets and surveys are also ideally placed to monitor for invasive species. Warming polar regions are gateways for invasive species from lower latitudes, especially if these non-native species can adapt to the local conditions prevailing in warming polar ecosystems. With polar seaways opening up, particularly the North-West Passage in the Arctic, and the warming of the sub-Antarctic, the introduction of highly competitive non-native invasive species via shipping is a real threat to polar marine biodiversity^77,78^. In the terrestrial realm, whilst the connectedness of the Arctic has long been an acknowledged problem for the introduction of non-native invasives, increased human activities in Antarctica are also providing threats^79^. If these activities inadvertently introduce new species that out-compete native species, or equally likely, new diseases and parasites emerge or change their host preferences, this could result in widespread ecosystem restructuring and native species extinctions^80^. Genomic screening not only offers the possibility of identifying populations under stress and changing ecosystems well before other approaches, but it can also be used for the monitoring of invasive species, thereby facilitating early interventions. Long-term monitoring sites are a critical resource to underpin surveillance activities.

Most national polar science programmes conduct some form of long-term monitoring, usually operating out of established research stations. Examples include the Rothera Time Series (RaTS, https://www.bas.ac.uk/project/rats/, since 1997) on the Antarctic Peninsula; the Long Term Ecological Research Network (LTER) Hausgarten Observatory in the Atlantic-Arctic gateway of the Fram Strait (since 1999); the McMurdo Dry Valleys LTER program (https://mcm.lternet.edu/) since 1992; and various stations in the Arctic (https://arc-lter.ecosystems.mbl.edu), with some station measurements dating back to 1987. There are also long-term ship-based surveys such as the Palmer LTER (https://pallter.marine.rutgers.edu) which has been running since 1992 on the Antarctic Peninsula. These provide crucial real-time data on the magnitude and progression of climate change effects on ecosystems, while enabling seasonal effects and interannual “noise”, often from large environmental cycles such as El Niño or the Southern Annular mode (SAM), to be disentangled from longer-term climate-driven changes^26^. In addition, the polar regions contain numerous protected terrestrial sites, whilst to date, there are just three established Marine Protected Areas (MPAs) in the Southern Ocean (Antarctic Peninsula, Weddell Sea and East Antarctic), although at the moment we have no measures to assess their effectiveness.

#### State of the art

We are only aware of very few long-term studies that explicitly incorporate routine genetic sampling and analysis (e.g. Antarctic fur seals^12^). Tentative steps have been taken with multi-gene metabarcoding to generate biological baseline data for a handful of research stations^81^. However, these studies are somewhat limited in scope, focusing mainly on 16S rDNA and 18S rDNA amplicon sequencing with restricted sampling within and among seasons. In addition, some polar research stations have been involved in global initiatives employing standardised methodologies, such as the “Ocean Sampling Day” (OSD) (https://www.assembleplus.eu/research/ocean-sampling-day) and the global ARMS (Artificial Reef Monitoring Structures) initiative (https://naturalhistory.si.edu/research/global-arms-program). Another potentially useful tool that is being trialled for surveillance in the polar regions is environmental DNA (eDNA). Successful pilot studies have been carried out using shotgun metagenomics and amplicon sequencing in the West Antarctic Peninsula and Canadian Arctic ports respectively^82,83^. eDNA metabarcoding has also proven useful for detecting long-term changes in kittiwake diet linked to “Atlantification”^5,84^. Alongside the application of genomic technologies to monitoring and surveillance strategies, there is now the possibility to carry out sequencing on-site. In particular, the increasing availability of transportable sequencing devices, largely in the form of the MinION (https://nanoporetech.com), has enabled in-field sequencing laboratories to perform real-time environmental sequencing, even in the polar regions^85^.

#### Perspectives and recommendations

Accurate biodiversity surveillance requires long-term monitoring programmes, whether these are based on research stations or regular surveys. These activities are imperative in areas of the planet that are either remote and difficult to monitor or which are changing rapidly. The polar regions are both, so they require the highest priority for future actions. Therefore, long-term monitoring programmes, including genomic based biodiversity assessments, in the polar regions should not only be strongly supported, but strategic planning is required to implement more surveys in critical areas, such as protected areas. Global initiatives such as OSD and ARMS should be incorporated more widely into polar research programmes, both to improve the amount and standardisation of data gathered from polar regions, and to better understand polar ecosystems in a global context. Similar global initiatives for terrestrial ecosystems should be extended polewards. Further work is also required to standardise eDNA analyses for surveillance. Many studies to date use metabarcoding (amplicon) approaches, which are limited due to PCR bias^86^. More extensive genetic analyses should therefore be employed, such as metagenomics^82^ which could, depending on sequencing depth, generate whole genomes (e.g., metagenome assembled genomes) or mitogenomes with increased taxonomic resolution linked to their functional potential. On the technology front, real time DNA sampling and analysis should ideally be integrated with autonomous collecting systems (e.g., moored remote access samplers or Autonomous Underwater Vehicles (AUVs)). Prototypes are being developed that are currently limited to automatic sample acquisition in temperate marine and freshwater environments^87,88^. Similar trials exist for AUVs^89^. More specialised applications being developed include chemically synthesised graphene oxide nanosheets for monitoring red tides, microfluidic devices for label-free DNA detection^90^ and combining eDNA technologies with remote sensing^91^. As these prototype collection technologies become more robust and standardised, they will also likely be modified and trialled for the rigours of the polar environment, alongside integrated DNA sequencing, so that real-time DNA data can be sent directly to the researcher, allowing information to be more readily acquired from difficult to access and therefore data poor regions. Overall there is a need to drive the use of cutting edge genomic technologies into polar biodiversity sciences.

### Polar Genomics: a road map

With the polar regions of our planet under threat, there is an imperative to obtain full genome sequences for diverse organisms inhabiting polar ecosystems, from the deep oceans to the permafrost on land, for both the Arctic and Antarctic. This will enable the wider application of omics technologies to polar species, which will improve our understanding of evolution in the cold and adaptive responses to a warming world. More broadly, active and wider promotion of polar biology, including emphasis of the economic benefits, should bring novel interdisciplinary collaborations and also push the research to the fore with funding bodies. Being viewed as interdisciplinary and less polar-centric should increase funding possibilities, especially if polar research communities organise themselves into consortia to lobby for funding and take advantage of funding calls, including international opportunities. The polar community also needs to engage more widely, at all levels from the general public to funding bodies and governments, to lobby and convince them of the scientific and economic benefits that investment in polar biological research will bring. These benefits include conserving polar biodiversity for future generations, maintaining crucial polar ecosystem services, and the sustainable exploitation of polar organisms for societal gain.

To achieve these goals under a currently constrained funding-limited landscape, we call for a polar initiative similar in scope to global programmes such as “The Darwin Tree of Life Project” (https://www.darwintreeoflife.org/) and the “EarthBiogenome Project” (https://www.earthbiogenome.org/). As polar researchers, we need:

- Stronger engagement with the wider scientific community and actively expand engagement by those outside the traditional polar community by increased attendance and presentations at non-polar conferences. This could be achieved by, for example, organising polar biology related symposia within broader non-polar conferences such those organised by the Society for Experimental Biology and ecological societies, or presenting polar data at specialised conferences on, for example, biomineralization or genomics.
- To identify the big fundamental questions in ecology and how we can answer them using polar species and omics approaches.
- To increase the value of polar research by conducting more comparative work in different ecosystems, linking polar biology to the context of biology world-wide.
- To engage with the big genome centres on the importance of sequencing polar species and lobby for funding.
- To develop polar model species by importing functional analyses from non-polar model species, particularly to understand cold adapted evolution.
- To emphasise the economic benefits which can emerge from polar biological studies, and increase public awareness of life in polar ecosystems and how it contributes to the wellbeing of humankind. For example, use the next International Polar Year to advertise polar science to the wider community.
- To engage with governments, philanthropies, and industrial stakeholders to raise awareness that polar life forms are worth protecting, archiving (e.g., gene banks) and sustainably exploiting due to biological novelty not found in other ecosystems on Earth.

### Supplementary information

Supplementary Information
