# Supplementary material for: Multi-omics for studying and understanding polar life
Source: Nat Commun. 2023 Nov 17;14:7451. doi: 10.1038/s41467-023-43209-y (PMC10656552; doi:10.1038/s41467-023-43209-y)
Supplement: Supplementary file 1 — Supplementary Information [file 41467_2023_43209_MOESM1_ESM.pdf]

Supplementary material

Multi-omics for studying and understanding polar life

Authors: Clark MS[1*], Hoffman JI[1,2*], Peck LS[1*], Bargelloni L[3], Gande, D[4], Havermans C[5], Meyer B[5,6,7], Patarnello T[3], Phillips T[1], Stoof-Leichsenring KR[8,] Vendrami DLJ[2], Beck A[9], Collins G[10,11], Friedrich MW[4], Halanych, KM[12], Masello JF[2,13], Nagel R[2,14], Norén K[15], Printzen C[10,16], Ruiz MB[5,17], Wohlrab S[5,7], Becker B[18], Dumack K[19], Ghaderiardakani F[20], Glaser K[21], Heesch S[21], Held C[5], John U[5], Karsten U[21], Kempf S[5], Lucassen M[5], Paijmans A[2], Schimani K[22], Wallberg A[23], Wunder LC[4], Mock T[24*].

Affiliations:
[1]British Antarctic Survey, UKRI-NERC, High Cross, Madingley Road, Cambridge, CB3 0ET, UK.
[2]Universität Bielefeld, VHF, Konsequenz 45, 33615 Bielefeld, Germany.
[3]Università degli Studi di Padova, Dept. of Comparative Biomedicine and Food Science, viale dell'Università 16, I-35020 Legnaro, Italy.
[4]Microbial Ecophysiology group, Faculty of Biology/Chemistry & MARUM, University of Bremen, Leobener Straße 3, 28359 Bremen, Germany.
[5]Alfred-Wegener-Institut Helmholtz-Zentrum für Polar- und Meeresforschung, Am Handelshafen 12, 27570 Bremerhaven, Germany.
[6]Institute for Chemistry and Biology of the Marine Environment, University of Oldenburg, Oldenburg, Germany.
[7]Helmholtz Institute for Functional Marine Biodiversity at the University of Oldenburg (HIFMB), 23129 Oldenburg, Germany.
[8]Alfred-Wegener-Institute Helmholtz Centre for Polar and Marine Research, 14473, Potsdam, Germany.
[9]Staatliche Naturwissenschaftliche Sammlungen Bayerns, Botanische Staatssammlung München (SNSB-BSM), Menzinger Str. 67, 80638 München, Germany.
[10]Senckenberg Biodiversity and Climate Research Centre & Loewe-Centre for Translational Biodiversity Genomics, Senckenberganlage 25, 60325 Frankfurt am Main, Germany.
[11]Current address: Manaaki Whenua - Landcare Research, 231 Morrin Road St Johns Auckland 1072, New Zealand.
[12]Center for Marine Science, University of North Carolina, 5600 Marvin K. Moss Lane Wilmington, NC 28409 Wilmington, USA.
[13]Justus-Liebig-Universität Gießen, Germany.
[14]Current address: University of St Andrews, School of Biology, St Andrews, Fife, KY16 9TH, UK.
[15]Stockholm University, Department of Zoology, 106 91 Stockholm, Sweden.
[16]Natural History Museum Frankfurt, Senckenberganlage 25, 60325 Frankfurt am Main, Germany.
[17]Universität Duisburg-Essen, Universitätstrasse 5, 45151 Essen, Germany.
[18]Universität zu Köln, Institut für Pflanzenwissenschaften, Zülpicher Str. 47b, 60674 Köln, Germany.
[19]Universität zu Köln, Terrestrische Ökologie, Zülpicher Str. 47b, 60674 Köln, Germany.
[20]Institute for Inorganic and Analytical Chemistry, Friedrich Schiller University Jena, Lessingstraße 8, 07743 Jena, Germany.
[21]Institute of Biological Sciences, Applied Ecology and Phycology, University of Rostock, Albert-Einstein-Straße 3, 18059 Rostock.
[22]Botanischer Garten und Botanisches Museum Berlin, Freie Universität Berlin, Königin-Luise-Straße 6-8, 14195 Berlin, Germany.
[23]Uppsala University, Department of Medical Biochemistry and Microbiology, Husargatan 3, 751 23 Uppsala, Sweden.
[24]School of Environmental Sciences, University of East Anglia, Norwich Research Park, NR4 7TJ, Norwich, UK.

Corresponding authors:
Clark MS: British Antarctic Survey, UKRI-NERC, Madingley Road, Cambridge, CB3 0ET, UK. Email: mscl@bas.ac.uk
Hoffman JI: Department of Animal Behaviour, University of Bielefeld, Bielefeld, Germany. Email: joseph.hoffman@uni-bielefeld.de and British Antarctic Survey, UKRI-NERC, Madingley Road, Cambridge, CB3 0ET, UK.
Peck, LS: British Antarctic Survey, UKRI-NERC, Madingley Road, Cambridge, CB3 0ET, UK. Email: lspe@bas.ac.uk
Mock, T: School of Environmental Sciences, University of East Anglia, Norwich Research Park, Norwich, NR4 7TJ, UK. Email: t.mock@uea.ac.uk

Supplementary material:

Supplementary Note 1
Supplementary Note 2

Supplementary Note 1: Supplementary figure detailing 21st century projected change in temperature in the poles and tables of area of regions with annual mean 2m air temperature below -20 °C, -10 °C or 0 °C to supplement figure and table of CMIP6 model ensemble members whose mean is used to show the areas of regions below 20 °C, -10 °C or 0 °C. Full references are provided at the end of the supplemental information.

Figure showing 21st century projected change in temperature in the poles. Polar-centred Lambert Azimuthal Equal Area plots for the Northern and Southern hemispheres (showing from the pole to 60° North or South). Regions with annual mean 2m air temperatures below -20°C (dark blue), between -20°C and -10°C (mid-blue), between -10°C and 0°C (pale blue) and above 0°C (white). Maps are shown for CMIP6 ScenarioMIP experiments SSP1-2.6, SSP2-4.5 and SSP3-7.0, which have total radiative forcing levels by the end of the 21st century of ~2.6 W m-2, ~4.5 W m-2 and ~7.0 W m-2 respectively[1]. The ensemble contains one run of each CMIP6 model or model physics variant available in the CEDA CMIP6 archive (https://catalogue.ceda.ac.uk/uuid/b96ce180077f4810abc4eef0e48901d9) for which monthly 2m air temperature data were available for all of experiments SSP1-2.6, SSP2-4.5 and SSP3-7.0 and which have a transient climate response in the 'likely' (66% likelihood) range 1.4-2.2 °C, as suggested[2].

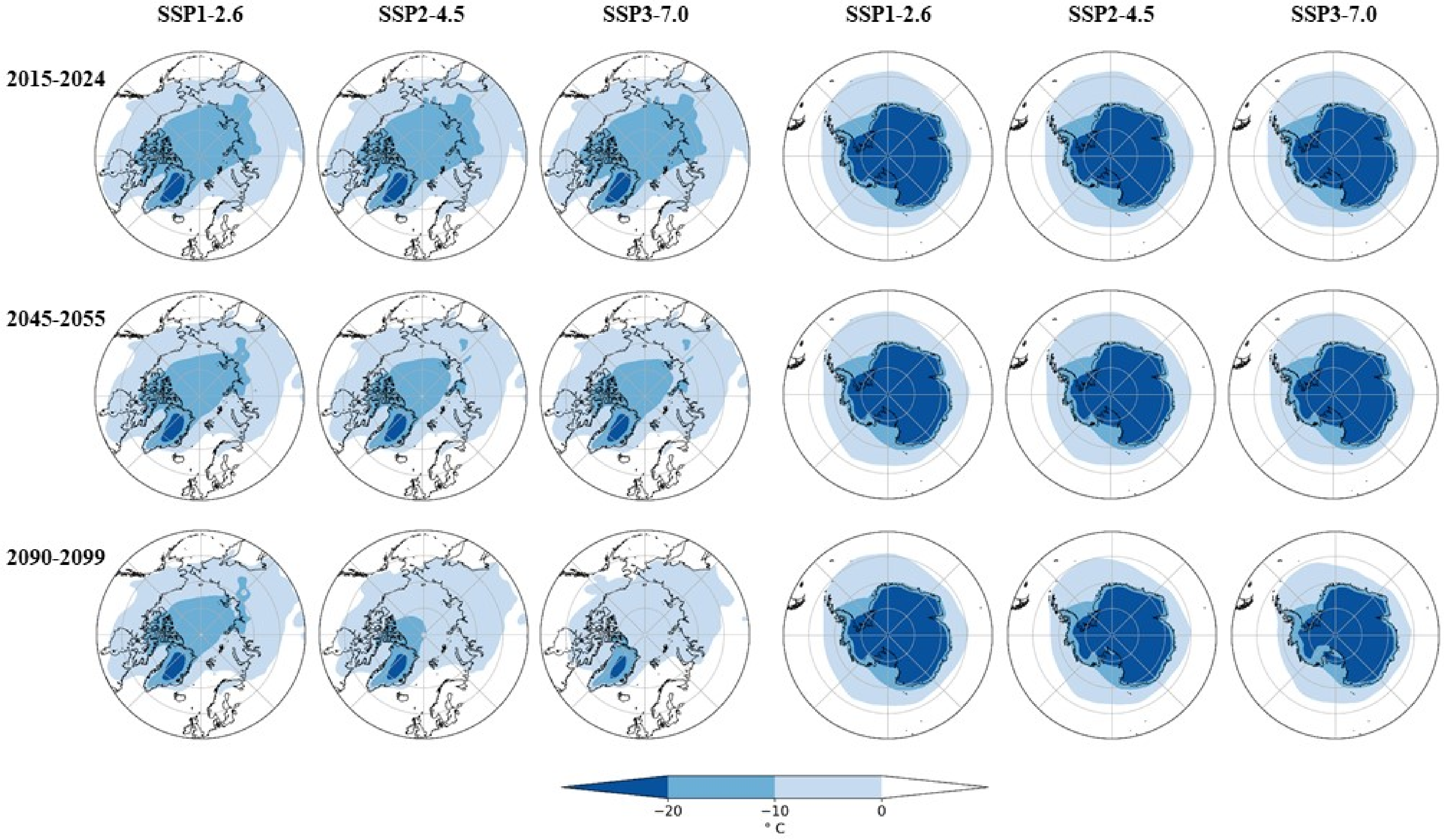
SSP1-2.6
SSP2-4.5
SSP3-7.0
SSP1-2.6
SSP2-4.5
SSP3-7.0
2015-2024
2045-2055
2090-2099
−20
−10
0
° C

Table of area of regions with annual mean 2m air temperature below -20 °C, -10 °C or 0 °C to supplement figure above. Models used were CMIP6 (coupled model intercomparison project, phase 6) ScenarioMIP[1] runs with forcings from different SSP (shared socioeconomic pathway) scenarios[3].

SSP3-7.0 is the baseline (unmitigated by climate policy) outcome of Integrated Assessment Models (IAMs) driven by the SSP3 (regional rivalry) narrative[4] in which energy production is dominated by fossil fuels (especially coal – see fig 3b in the above) and which has "particularly high aerosol emissions and land use change": see page 3469[1] and is thus a high emissions scenario. SSP2-4.5 is the result of climate policies mitigating the baseline consequences of the SSP2 (middle of the road) narrative to reduce end-of-21st century radiative forcing from 6.5 Wm^{-2} to 4.5 Wm^{-2} (see table 2[5]) and therefore is much nearer the middle of the range of projected radiative forcings. SSP1-2.6 is a low emissions scenario that is the result of mitigation reducing the baseline consequences of the SSP1 (sustainability) narrative to reduce the end-of-21st century radiative forcing from around 5 Wm^{-2} to 2.6 Wm^{-2} (as in[6]).

**Temperature threshold**	**Time period**	**Experiment**		
		**SSP1-2.6**	**SSP2-4.5**	**SSP3-7.0**
**Area of region below -20 °C (million km²)**	Current conditions (2015-2024 mean)	12.4	12.3	12.4
	Middle of 21st Century (2045-2055 mean)	12.1	12.0	11.9
	End of 21st Century (2090-2099 mean)	12.0	11.4	10.6
**Area of region below -10 °C (million km²)**	Current conditions (2015-2024 mean)	30.0	30.4	30.5
	Middle of 21st Century (2045-2055 mean)	26.6	24.4	23.9
	End of 21st Century (2090-2099 mean)	25.4	19.0	16.4
**Area of region below 0 °C (million km²)**	Current conditions (2015-2024 mean)	70.7	70.7	70.8
	Middle of 21st Century (2045-2055 mean)	66.3	64.9	63.9
	End of 21st Century (2090-2099 mean)	65.4	58.8	51.8

Table of CMIP6 model ensemble members whose mean is used to show the areas of regions below -20 °C, -10 °C or 0 °C in the table above.

Institution	Model	Ensemble member	Data source and reference
AWI	AWI-CM 1.1 MR	r1i1p1f1	https://doi.org/10.22033/ESGF/CMIP6.376[7]
BCC	BCC-CSM2 MR	r1i1p1f1	https://doi.org/10.22033/ESGF/CMIP6.1732[8]
CAMS	CAMS-CSM 1.0	r1i1p1f1	https://doi.org/10.22033/ESGF/CMIP6.11004[9]
CAS	FGOALS-f3-L	r1i1p1f1	https://doi.org/10.22033/ESGF/CMIP6.2046[10]
	FGOALS-g3	r1i1p1f1	https://doi.org/10.22033/ESGF/CMIP6.2056[11]
CCCR-IITM	IITM-ESM	r1i1p1f1	https://doi.org/10.22033/ESGF/CMIP6.14741[12]
CMCC	CMCC-CM2-SR5	r1i1p1f1	https://doi.org/10.22033/ESGF/CMIP6.1365[13]
	CMCC-ESM2	r1i1p1f1	https://doi.org/10.22033/ESGF/CMIP6.13168[14]
CNRM-CERFACS	CNRM-ESM2-1	r1i1p1f2	https://doi.org/10.22033/ESGF/CMIP6.1395[15]
CSIRO	ACCESS-ESM1.5	r1i1p1f1	https://doi.org/10.22033/ESGF/CMIP6.2291[16]
CSIRO-ARCCSS	ACCESS-CM2	r1i1p1f1	https://doi.org/10.22033/ESGF/CMIP6.2285[17]
INM	INM-CM5-0	r1i1p1f1	https://doi.org/10.22033/ESGF/CMIP6.12322[18]
MIROC	MIROC6	r1i1p1f1	https://doi.org/10.22033/ESGF/CMIP6.898[19]
	MIROC-ES2L	r1i1p1f2	https://doi.org/10.22033/ESGF/CMIP6.936[20]
MPI-M	MPI-ESM1.2-LR	r1i1p1f1	https://doi.org/10.22033/ESGF/CMIP6.793[21]
MRI	MRI-ESM2.0	r1i1p1f1	https://doi.org/10.22033/ESGF/CMIP6.638[22]
NASA-GISS	GISS-E2.1G	r1i1p1f2	https://doi.org/10.22033/ESGF/CMIP6.2074[23]
		r1i1p3f1	
NCAR	CESM2	r4i1p1f1	https://doi.org/10.22033/ESGF/CMIP6.2201[24]
	CESM2-WACCM	r1i1p1f1	https://doi.org/10.22033/ESGF/CMIP6.10026[25]
NCC	NorESM2-LM	r1i1p1f1	https://doi.org/10.22033/ESGF/CMIP6.604[26]
NIMS-KMA	KACE1.0-G	r1i1p1f1	https://doi.org/10.22033/ESGF/CMIP6.2242[27]
NOAA-GFDL	GFDL-ESM4	r1i1p1f1	https://doi.org/10.22033/ESGF/CMIP6.9242[28]
UA	MCM-UA-1-0	r1i1p1f2	https://doi.org/10.22033/ESGF/CMIP6.13816[29]

References for Supplementary Note 1

1. O'Neill B.C. et al. The Scenario Model Intercomparison Project (ScenarioMIP) for CMIP6, Geoscientific Model Development. 9, 3461–3482 (2016).
2. Hausfather Z. et al. Climate simulations: recognize the 'hot model' problem. Nature 605, 26-29 (2022).
3. Meinshausen M. et al. The shared socio-economic pathway (SSP) greenhouse gas concentrations and their extensions to 2500. Geoscientific Model Development. 13, 3571-3605 (2020).

4. Riahi K. et al. The Shared Socioeconomic Pathways and their energy, land use, and greenhouse gas emissions implications: An overview. Global Environmental Change. 42, 153-168 (2017).
5. Fricko O. et al. The marker quantification of the Shared Socioeconomic Pathway 2: A middle-of-the-road scenario for the 21st century. Global Environmental Change. 42, 251-267 (2017).
6. Van Vuuren D.P. et al. The Shared Socio-economic Pathways: Trajectories for human development and global environmental changeGlobal Environmental Change. 42, 237-250 (2017).
7. Semmler T. et al. AWI AWI-CM1.1MR model output prepared for CMIP6 ScenarioMIP. Version 20190529. Earth System Grid Federation (2019).
8. Xin X. et al. BCC BCC-CSM2MR model output prepared for CMIP6 ScenarioMIP. Version 20190314. Earth System Grid Federation (2019).
9. Rong X. CAMS CAMS-CSM1.0 model output prepared for CMIP6 ScenarioMIP. Version 20190708. Earth System Grid Federation (2019).
10. Yu Y. CAS FGOALS-f3-L model output prepared for CMIP6 ScenarioMIP. Version 20191013. Earth System Grid Federation (2019).
11. Li L. CAS FGOALS-g3 model output prepared for CMIP6 ScenarioMIP. Version 20200927 (SSP1-2.6), 20190818 (SSP2-4.5), 20190820 (SSP3-7.0). Earth System Grid Federation (2019).
12. Panickal S. et al. CCCR-IITM IITM-ESM model output prepared for CMIP6 ScenarioMIP. Version 20201112 (SSP1-2.6), 20200915 (SSP2-4.5, SSP3-7.0). Earth System Grid Federation (2020).
13. Lovato T. & Peano D. CMCC CMCC-CM2-SR5 model output prepared for CMIP6 ScenarioMIP. Version 20200717 (SSP1-2.6), 20200617 (SSP2-4.5), 20200622 (SSP3-7.0). Earth System Grid Federation (2020).
14. Lovato T., Peano D., Butenschön M. CMCC CMCC-ESM2 model output prepared for CMIP6 ScenarioMIP. Version 20210126 (SSP1-2.6), 20210129 (SSP2-4.5), 20210202 (SSP3-7.0). Earth System Grid Federation (2021).
15. Seferian R. CNRM-CERFACS CNRM-ESM2-1 model output prepared for CMIP6 ScenarioMIP. Version 20190328 (SSP1-2.6, SSP2-4.5), 20191021 (SSP3-7.0). Earth System Grid Federation (2019).
16. Ziehn T. et al. CSIRO ACCESS-ESM1.5 model output prepared for CMIP6 ScenarioMIP. Version 20210318 (SSP1-2.6), 20131115 (SSP2-4.5, SSP3-7.0). Earth System Grid Federation (2019).
17. Dix M. et al. CSIRO-ARCCSS ACCESS-CM2 model output prepared for CMIP6 ScenarioMIP. Version 20210317 (SSP1-2.6), 20191108 (SSP2-4.5, SSP3-7.0). Earth System Grid Federation (2019).
18. Volodin E. et al. INM INM-CM5-0 model output prepared for CMIP6 ScenarioMIP. Version 20190619 (SSP1-2.6, SSP2-4.5), 20190618 (SSP3-7.0). Earth System Grid Federation (2019).
19. Shiogama H., Abe M., Tatebe H. MIROC MIROC6 model output prepared for CMIP6 ScenarioMIP. Version 20190627. Earth System Grid Federation (2019).
20. Tachiiri K. et al. MIROC MIROC-ES2L model output prepared for CMIP6 ScenarioMIP. Version 20220314 (SSP1-2.6), 20190823 (SSP2-4.5, SSP3-7.0). Earth System Grid Federation (2019).
21. Wieners K-H. et al. MPI-M MPIESM1.2-LR model output prepared for CMIP6 ScenarioMIP. Version 20190710. Earth System Grid Federation (2019).
22. Yukimoto S. et al. MRI MRI-ESM2.0 model output prepared for CMIP6 ScenarioMIP. Version 20191108 (SSP1-2.6), 20190222 (SSP2-4.5, SSP3-7.0). Earth System Grid Federation (2019).

23. NASA Goddard Institute for Space Studies (NASA/GISS). NASA-GISS GISS-E2.1G model output prepared for CMIP6 ScenarioMIP. Version 20200115. Earth System Grid Federation (2020).
24. Danabasoglu G. NCAR CESM2 model output prepared for CMIP6 ScenarioMIP. Version 20200528. Earth System Grid Federation (2019a).
25. Danabasoglu G. NCAR CESM2-WACCM model output prepared for CMIP6 ScenarioMIP. Version 20210211 (SSP1-2.6), 20190815 (SSP2-4.5, SSP3-7.0). Earth System Grid Federation (2019b)
26. Seland Ø. et al. NCC NorESM2-LM model output prepared for CMIP6 ScenarioMIP. Version 20191108. Earth System Grid Federation (2019).
27. Byun Y-H. et al. NIMS-KMA KACE1.0-G model output prepared for CMIP6 ScenarioMIP. Version 20191007 (SSP1-2.6), 20191217 (SSP2-4.5, SSP3-7.0). Earth System Grid Federation (2019)
28. Guo H. et al. NOAA-GFDL GFDL-CM4 model output prepared for CMIP6 ScenarioMIP. Version 20180701. Earth System Grid Federation (2018).
29. Stouffer R. UA MCM-UA-1-0 model output prepared for CMIP6 ScenarioMIP. Version 20190731. Earth System Grid Federation (2019).

Supplemental Note 2

Full description of photographs and acknowledgements of photographs used in Figure 2 with references relating to the polar organisms' unique adaptations.

A) **Polar bears** (*Ursus maritimus*) have evolved a modified cardiovascular system that allows them to tolerate chronically elevated levels of serum cholesterol in their diet[1]. Photographed on sea ice in the Fram Straight 79° north Norwegian Cruise. Photo from BAS photo library. Photographer Angelika Renner.

B) **Antarctic sea spider** *Decolopoda australis* grows to around 30 cm from tip to tip and is an example of polar gigantism[2]. Furthermore, there are over twice the number of species of pycnogonids in the Antarctic compared with the global average[3]. Photo from BAS photo library. Photographer Dave Bowden.

C) **Diatom** (*Fragilariopsis cylindrus*) produces numerous ice antifreeze proteins to allow it to survive in sea ice[4,5]. It is a good candidate model species for which transformation technologies are already available[6]. Photograph is Scanning Electron Microscope (SEM) image courtesy of Thomas Mock, University of East Anglia, UK.

D) **Copepod** (*Calanus finmarchicus*) massively accumulates lipids (up to 70% of individual dry weight) to enable it to survive at high latitudes by entering a winter resting state or diapause[7,8]. Photograph courtesy of Kim Last, Scottish Association for Marine Sciences, UK.

E) **Antarctic springtail** (*Cryptopygus antarcticus*) at 1-2mm long is the largest permanently land dwelling organism in Antarctica, surviving down to -30°C using a process called rapid cold hardening[9]. Phylogenetic analysis has proved that it survived in refugia during glaciation events in Antarctica, confounding previous reconstructions of Antarctic glacial history[10]. Photograph is a false colour, scanning electron-micrograph. Photo from BAS photo library. Photographer Pete Bucktrout.

F) **Icefish** (*Chionodraco hamatus*) is one of the 16 species belonging to the family Channichthyidae, which are the only vertebrates that lack haemoglobin. They survive on diffused oxygen from the surrounding sea water. Footprints of haemoglobin genes remain in these species' genomes, but are dramatically deleted and non-functional[11,12]. This evolutionary trait is also accompanied by other cardiac modifications such as larger hearts and blood vessels[13]. Photograph courtesy of Gianfranco Santovito, University of Padua.

G) **Endolithic microbial communities are present as blue coloured bands in the rock.** Close-up of rock containing endolithics from by Lake Hoare, Antarctic Dry Valleys[14]. These communities survive the most extreme conditions with very low cell division rates of 4-38 per year. This microniche is proposed to protect the community from desiccation, rapid temperature variation and UV radiation flux whilst still allowing penetration of photosynthetically active radiation for use by phototrophs[15,16]. Photo from BAS photo library. Photographer David Wynn-Williams.

H) **Krill (***Euphausia superba*) has the largest biomass of any wild animal on the planet (300-500 million tonnes), critically underpinning the Southern Ocean Ecosystem. Historic sampling has revealed shifts in distributions southwards over the past 100 years due to climate change[17]. It also has an enormous genome around 15x that of humans[18]. Photo from BAS photo library. Photographer Pete Bucktrout.

I) **Arctic tern** (*Sterna paradisaea*) undertakes the longest migration on Earth with an annual circuit of 50,000km[19]. Photo from BAS photo library. Photographer Callan Duck.

J) **Antarctic nematode** (*Eudorylaimus sp.*) is a terrestrial omnivore apex predator which normally lives in temperatures of down to -7°C along the Antarctic Peninsula, although some nematodes can survive several years at -80°C and very low desiccation levels[20]. All survive substantial and rapid temperature changes due to diurnal freeze-thaw cycles[21,22]. Photograph courtesy of Kevin Newsham, British Antarctic Survey, UK

K) **Arctic bell-heather** (*Cassiope tetragona*) is a dwarf shrub found in Arctic tundra, that thrives in deep snow over winter[23] and whose annual growth patterns can be used as a proxy for Arctic climate[24]. Photograph courtesy of Melody Clark, British Antarctic Survey, UK

L) **Polar cod** (*Boreogadus saida*) survives the cold with a structurally identical antifreeze glycoprotein to Antarctic fish[25]. However, both molecules have very different evolutionary origins, providing a classical example of convergent evolution. Retreating Arctic sea ice is changing the distribution of this species[26]. Photograph courtesy of Till Luckenbach, Helmholtz Centre for Environmental Research - UFZ, Germany.

M) **Arctic intertidal blue mussels and macroalgae** (*Mytilus edulis* and *Ascophyllum nodosum*) thrive in the Greenland intertidal region, even when summer temperatures reach 36°C[27,28]. Photograph courtesy of Jakob Thyrring, Aarhus University, Denmark.

N) **Ectomycorrhiza** Root of the Arctic plant species *Bistorta vivipara* colonised by an ectomycorrhizal fungus. Ectomycorrhizal symbioses have vital roles in the uptake of nutrients from soil by roots and help protect the plant from extreme conditions[29]. They are absent from the Antarctic. Photograph courtesy of Kevin Newsham, British Antarctic Survey, UK.

O) **Ocean quahog** (*Arctica islandica*) holds the record of the longest-lived animal on Earth, with one famous individual "Hafrun" (in the photograph) established to have lived for 507 years in the wild[30,31]. Studies have shown very stable levels of antioxidants over 10s to100s of years[32]. Photograph courtesy of Al Wanamaker, Iowa State University, USA.

P) **Antarctic fur seal** (*Arctocephalus gazella*) population genomics is revealing signals of past hunting pressures, shedding light on community level impacts and ecosystem disruption[33,34]. Photograph courtesy of Joseph Hoffman, University of Bielefeld, Germany.

References for Supplementary Note 2

1. Liu, S.P. et al. Population genomics reveal recent speciation and rapid evolutionary adaptation in polar bears. Cell 157, 785-794 (2014).
2. Chapelle, G. & Peck, L.S. Polar gigantism dictated by oxygen availability. Nature 399, 114-115 (1999).
3. Clarke, A. & Johnston, N.M. Antarctic marine benthic diversity. in Oceanography and Marine Biology. Volume 41, Vol. Volume 41 (eds. Gibson, R.N. & Atkinson, R.J.A.) 47-114 (2003).
4. Bayer-Giraldi, M., Uhlig, C., John, U., Mock, T. & Valentin, K. Antifreeze proteins in polar sea ice diatoms: diversity and gene expression in the genus Fragilariopsis. Environmental Microbiology 12, 1041-1052 (2010).
5. Mock, T. et al. Evolutionary genomics of the cold-adapted diatom Fragilariopsis cylindrus. Nature 541, 536-540 (2017).
6. Faktorova, D. et al. Genetic tool development in marine protists: Emerging model organisms for experimental cell biology. Nature Methods 17, 481-494 (2020).

7. Tarling, G.A. et al. Carbon and Lipid Contents of the Copepod *Calanus finmarchicus* Entering Diapause in the Fram Strait and Their Contribution to the Boreal and Arctic Lipid Pump. Frontiers in Marine Science 9, https://doi.org/10.3389/fmars.2022.926462 (2022).
8. Coguiec, E. et al. Photoperiodism and overwintering in boreal and sub-Arctic *Calanus finmarchicus* populations. Marine Ecology Progress Series 712, 49-65 (2023).
9. Worland, M.R. & Convey, P. Rapid cold hardening in Antarctic microarthropods. Functional Ecology 15, 515-524 (2001).
10. Convey, P. et al. Antarctic terrestrial life - challenging the history of the frozen continent? Biological Reviews 83, 103-117 (2008).
11. Cocca, E. et al. Genomic remnants of alpha-globin genes in the hemoglobinless Antarctic icefishes. Proceedings of the National Academy of Sciences of the United States of America 92, 1817-1821 (1995).
12. Bargelloni, L., Marcato, S., Patarnello, T. Antarctic fish hemoglobins: Evidence for adaptive evolution at subzero temperature. Proceedings of the National Academy of Sciences of the United States of America, 95, 670-8675 (1998).
13. Sidell, B.D. & O'Brien, K.M. When bad things happen to good fish: the loss of hemoglobin and myoglobin expression in Antarctic icefishes. Journal of Experimental Biology 209, 1791-1802 (2006).
14. Friedmann, E.I. Endolithic microorganisms in the Antarctic cold desert. Science 215, 1045-1053 (1982).
15. Hughes, K.A. & Lawley, B. A novel Antarctic microbial endolithic community within gypsum crusts. Environmental Microbiology 5, 555-565 (2003).
16. Sajjad, W. et al. Endolithic microbes of rocks, their community, function and survival strategies. International Biodeterioration & Biodegradation 169, 105387 (2022).
17. Atkinson, A., Siegel, V., Pakhomov, E. & Rothery, P. Long-term decline in krill stock and increase in salps within the Southern Ocean. Nature 432, 100-103 (2004).
18. Shao, C. et al. The enormous repetitive Antarctic krill genome reveals environmental adaptations and population insights. Cell 186, 1279-1294 (2023).
19. Alerstam, T., Bckman, J., Grnroos, J., Olofsson, P. & Strandberg, R. Hypotheses and tracking results about the longest migration: The case of the arctic tern. Ecology and Evolution 9, 9511-9531 (2019).
20. Newsham, K.K., Maslen, N.R. & McInnes, S.J. Survival of Antarctic soil metazoans at -80 degrees C for six years. Cryoletters 27, 291-294 (2006).
21. Simmons, B.L. et al. Long-term experimental warming reduces soil nematode populations in the McMurdo Dry Valleys, Antarctica. Soil Biology & Biochemistry 41, 2052-2060 (2009).
22. Knox, M.A. et al. Impact of diurnal freeze-thaw cycles on the soil nematode *Scottnema lindsayae* in Taylor Valley, Antarctica. Polar Biology 39, 583-592 (2016).
23. Blok, D. et al. Deepened winter snow increases stem growth and alters stem delta C-13 and delta N-15 in evergreen dwarf shrub *Cassiope tetragona* in high-arctic Svalbard tundra. Environmental Research Letters 10, 044008 (2015).
24. Rozema, J. et al. Annual growth of *Cassiope tetragona* as a proxy for Arctic climate: developing correlative and experimental transfer functions to reconstruct past summer temperature on a millennial time scale. Global Change Biology 15, 1703-1715 (2009).
25. Zhuang, X., Yang, C., Murphy, K.R. & Cheng, C.H.C. Molecular mechanism and history of non-sense to sense evolution of antifreeze glycoprotein gene in northern gadids. Proceedings of the National Academy of Sciences of the United States of America 116, 4400-4405 (2019).
26. Huserbråten, M.B.O., Eriksen, E., Gjøsæter, H. & Vikebø, F. Polar cod in jeopardy under the retreating Arctic sea ice. Communications Biology 2, 407 (2019).

27. Thyrring, J., Blicher, M.E., Sorensen, J.G., Wegeberg, S. & Sejr, M.K. Rising air temperatures will increase intertidal mussel abundance in the Arctic. Marine Ecology Progress Series 584, 91-104 (2017).
28. Clark, M.S., Peck, L.S. & Thyrring, J. Resilience in Greenland intertidal Mytilus: The hidden stress defense. Science of the Total Environment 767, 144366 (2021).
29. Acuna-Rodriguez, I.S., Newsham, K.K., Gundel, P.E., Torres-Diaz, C. & Molina-Montenegro, M.A. Functional roles of microbial symbionts in plant cold tolerance. Ecology Letters 23, 1034-1048 (2020).
30. Eiríksson, J, Scourse, J.D., Butler, P.G., Reynolds, D.J. & Símonarson, L.A. Langlífar kúskeljar, skeljatímatal og ástand sjávar við Norðurland í þúsund ár. Náttúrufræðingurinn 87, 95-108 (2017).
31. Butler, P.G., Wanamaker, A.D., Scourse, J.D., Richardson, C.A. & Reynolds, D.J. Variability of marine climate on the North Icelandic Shelf in a 1357-year proxy archive based on growth increments in the bivalve *Arctica islandica*. Palaeogeography Palaeoclimatology Palaeoecology 373, 141-151 (2013).
32. Sosnowska, D. et al. A Heart That Beats for 500 Years: Age-Related Changes in Cardiac Proteasome Activity, Oxidative Protein Damage and Expression of Heat Shock Proteins, Inflammatory Factors, and Mitochondrial Complexes in *Arctica islandica*, the Longest-Living Noncolonial Animal. Journals of Gerontology Series A-Biological Sciences and Medical Sciences 69, 1448-1461 (2014).
33. Humble, E. et al. RAD Sequencing and a Hybrid Antarctic Fur Seal Genome Assembly Reveal Rapidly Decaying Linkage Disequilibrium, Global Population Structure and Evidence for Inbreeding. G3-Genes Genomes Genetics 8, 2709-2722 (2018).
34. Hoffman, J.I. et al. Demographic Reconstruction of Antarctic Fur Seals Supports the Krill Surplus Hypothesis. Genes 13, 541 (2022).
